# Assessment of Fibrinolysis in Sepsis Patients with Urokinase Modified Thromboelastography

**DOI:** 10.1371/journal.pone.0136463

**Published:** 2015-08-26

**Authors:** Mauro Panigada, Lucia Zacchetti, Camilla L’Acqua, Massimo Cressoni, Massimo Boscolo Anzoletti, Rossella Bader, Alessandro Protti, Dario Consonni, Armando D’Angelo, Luciano Gattinoni

**Affiliations:** 1 Department of Anesthesiology, Intensive Care and Emergency, Fondazione IRCCS Ca' Granda - Ospedale Maggiore Policlinico, Milan, Italy; 2 A. Bianchi Bonomi Hemophilia and Thrombosis Center, Fondazione IRCCS Ca' Granda - Ospedale Maggiore Policlinico, Milan, Italy; 3 Epidemiology Unit, Fondazione IRCCS Ca' Granda - Ospedale Maggiore Policlinico, Milan, Italy; 4 Coagulation Service and Thrombosis Research Unit, IRCCS Ospedale San Raffaele, Milan, Italy; Erasmus Medical Centre, NETHERLANDS

## Abstract

**Introduction:**

Impairment of fibrinolysis during sepsis is associated with worse outcome. Early identification of this condition could be of interest. The aim of this study was to evaluate whether a modified point-of-care viscoelastic hemostatic assay can detect sepsis-induced impairment of fibrinolysis and to correlate impaired fibrinolysis with morbidity and mortality.

**Methods:**

This single center observational prospective pilot study was performed in an adult Intensive Care Unit (ICU) of a tertiary academic hospital. Forty consecutive patients admitted to the ICU with severe sepsis or septic shock were included. Forty healthy individuals served as controls. We modified conventional kaolin activated thromboelastography (TEG) adding urokinase to improve assessment of fibrinolysis in real time (UK-TEG). TEG, UK-TEG, plasminogen activator inhibitor (PAI)-1, thrombin-activatable fibrinolysis inhibitor (TAFI), d-dimer, DIC scores and morbidity (rated with the SOFA score) were measured upon ICU admission. Logistic regression was used to calculate odds ratios (ORs) and 95% confidence intervals (95% CIs) of mortality at ICU discharge.

**Results:**

UK-TEG revealed a greater impairment of fibrinolysis in sepsis patients compared to healthy individuals confirmed by PAI-1. TAFI was not different between sepsis patients and healthy individuals. 18/40 sepsis patients had fibrinolysis impaired according to UK-TEG and showed higher SOFA score (8 (6–13) vs 5 (4–7), p = 0.03), higher mortality (39% vs 5%, p = 0.01) and greater markers of cellular damage (lactate levels, LDH and bilirubin). Mortality at ICU discharge was predicted by the degree of fibrinolysis impairment measured by UK-TEG Ly30 (%) parameter (OR 0.95, 95% CI 0.93–0.98, p = 0.003).

**Conclusions:**

Sepsis-induced impairment of fibrinolysis detected at UK-TEG was associated with increased markers of cellular damage, morbidity and mortality.

## Introduction

Sepsis is associated with hemostatic abnormalities ranging from subclinical activation of blood coagulation (hypercoagulability) to massive thrombin and fibrin formation with systemic clotting activation [1]. In its initial phase the hypercoagulability may be associated with hypofibrinolysis which can be considered as an attempt to compartimentalize the infectious focus. As the infection gets worse, these local protective mechanisms may spread systemically, resulting in disseminated intravascular coagulation (DIC) [2] [3]. Thrombosis in the microcirculation may lead to different consequences depending on their possible dissolution by a more or less intact fibrinolytic system. In a previous work carried out in a group of patients with severe sepsis and septic shock, we found that the coagulation and inflammatory response were activated in all patients but unrelated with amount of organ failure and outcome, conversely fibrinolysis was inhibited in only a fraction of patients and was impressively associated with morbidity and mortality [4]. A similar observation was found in larger studies in patients with ARDS where impairment of fibrinolysis was associated with worse outcome [5] [6] [7]. Traditionally, the two main markers used to quantify fibrinolysis are Plasminogen Activator Inhibitor 1 (PAI-1) and Thrombin-activatable Fibrinolysis Inhibitor (TAFI). These markers are elevated in sepsis and related to multi organ failure and mortality [8] [9] [10] [11] [12], however no real time information can be obtained by these tests which require an experienced laboratory and have long turnaround times. Viscoelastic hemostatic assays such as thromboelastography and thromboelastometry have been used to characterize septic coagulopathy [13–16], specifically both hyper and hypo-coagulability and hyper-fibrinolysis. Conversely, hypo-fibrinolysis cannot be easily detected and quantitative evaluation of the impairment remains a challenge. Therefore we thought that the implementation of a modified point of care method for fibrinolysis assessment (UKIFTEG—Urokinase induced fibrinolysis in thromboelastography) [17], could be of interest in the sepsis population.

The aims of this study therefore were: I. to verify the feasibility of assessing fibrinolysis at the bedside using a modified point-of-care global assay of hemostasis (Urokinase Kaolin activated Thromboelastography, UK-TEG); II. to confirm or disprove that the fibrinolysis abnormalities are not universally present in the sepsis population; III. to verify whether sepsis-induced impairment of fibrinolysis correlate with higher severity of disease and risk of death. Of note, while previous studies on septic patients did not show any benefit from the untargeted treatment of hypercoagulability and hyper-inflammation [18] [19] [20] [21] [22], a third way of approach i.e. the correction of the fibrinolysis in patient who shows this alteration may be of future interest. We wish to describe here the results we obtained in a sample of severe sepsis/septic shock patients.

## Materials and Methods

### Study Population

Prospective observational study. The study protocol and the informed-consent form were approved by the ethic committee of the University Hospital Fondazione IRRCS Ca’ Granda—Ospedale Maggiore Policlinico, written informed consent or deferred consent was obtained from each patient. We used a sample size of 40 per group (n = 80), on the basis of feasibility and precision of estimates to be used to design the main study [23].

From February 2013 to September 2013 all patients admitted to the Intensive Care Unit (ICU) of Ospedale Maggiore Policlinico of Milan were screened. Patients 18 years of age or older who met the clinical criteria for severe sepsis or septic shock [24], were enrolled in the study (sepsis patients). Exclusion criteria included known congenital or chronic coagulation disorders, the current use of extracorporeal circuits for the treatment of the disease (such as extracorporeal membrane oxygenation or continuous veno-venous hemofiltration), and administration of anti-platelets drugs. A control group of 40 healthy nurses and physicians employed in the ICU was chosen (healthy individuals group).

Blood samples for thromboelastography analysis were drawn within 24 hours from the diagnosis of severe sepsis/septic shock. At the same time we determined plasminogen activator inhibitor-1 (PAI-1), thrombin activatable fibrinolysis inhibitor (TAFI), d-dimer, platelet count, aPTT, PT, LDH, creatinine, bilirubin, serum lactate, central venous O_2_ saturation (ScvO_2_), Sequential Organ Failure Assessment (SOFA) score [25]. We calculated DIC scores according to the International Society for Thrombosis and Haemostasis (ISTH “overt” 2001) [26] and according to the Japanese Association for Acute Medicine (JAAM 2006) [27]. Age, sex, site of infection, ICU mortality were also recorded.

### Thromboelastography

TEG (TEG 5000 Thrombelastograph Hemostasis Analyzer System, Haemonetics Corporation, Braintree, MA, USA) is a point of care device that provides information about the viscoelastic properties of blood clot during formation and lysis [28]. TEG was performed at 37°C, using citrated whole blood. Blood samples was obtained using an arterial line in place of sepsis patients after discarding 5 ml of blood and collected in siliconized Vacutainer tube (4.5 ml blood mixed with 0.5 ml of 105 mM trisodium citrate). TEG was performed after drawing blood, activating 1 ml of citrated blood in a tube containing kaolin and gently inverted five times as suggested by the manufacturer. An aliquot (340 μl) was placed in a warmed heparinase cup in the TEG and recalcified with 0.2 M calcium chloride (20 μl).

### Urokinase Kaolin Activated Thromboelastography (UK-TEG)

Hypofibrinolysis cannot be easily detected by conventional TEG. To unmask this condition ex-vivo we implemented a modified thromboelastographic technique for fibrinolysis assessment [17] that requires the addition of urokinase (UK, Urochinasi Crinos 100000 U/2ml) to enhance fibrinolysis (UK-TEG). UK was reconstituted with 5 ml of NaCl 0.9%, and 4 μl of this solution was added to the citrated whole blood in the kaolin-containing tube before inverting the tube. The final concentration of UK in the cup was 80 IU/ml of blood. TEG and UK-TEG were performed using the same blood sample.

The following parameters were registered both for TEG and UK-TEG: the time to initiate clot formation r (reaction time, minutes) influenced by the coagulation factors; the angle between the baseline and the tangent to the tracing (α-angle, degrees) influenced by fibrinogen and measuring the rapidity of fibrin cross-linking; the maximal amplitude of the tracing curve (MA, mm) representing clot strength depending on platelet function and platelet–fibrin interaction; the reduction in amplitude of the tracing curve at 30 and 60 minutes after reaching MA (Ly30%, Ly60% respectively) representing fibrinolysis. All the aforementioned parameters were calculated by the software provided by TEG Analytical Software (TAS version 4.2 for Windows, Haemonetics Corporation, Braintree, MA, USA).

### Preliminary Assessment of UK-TEG Procedure

**Fig 1:**
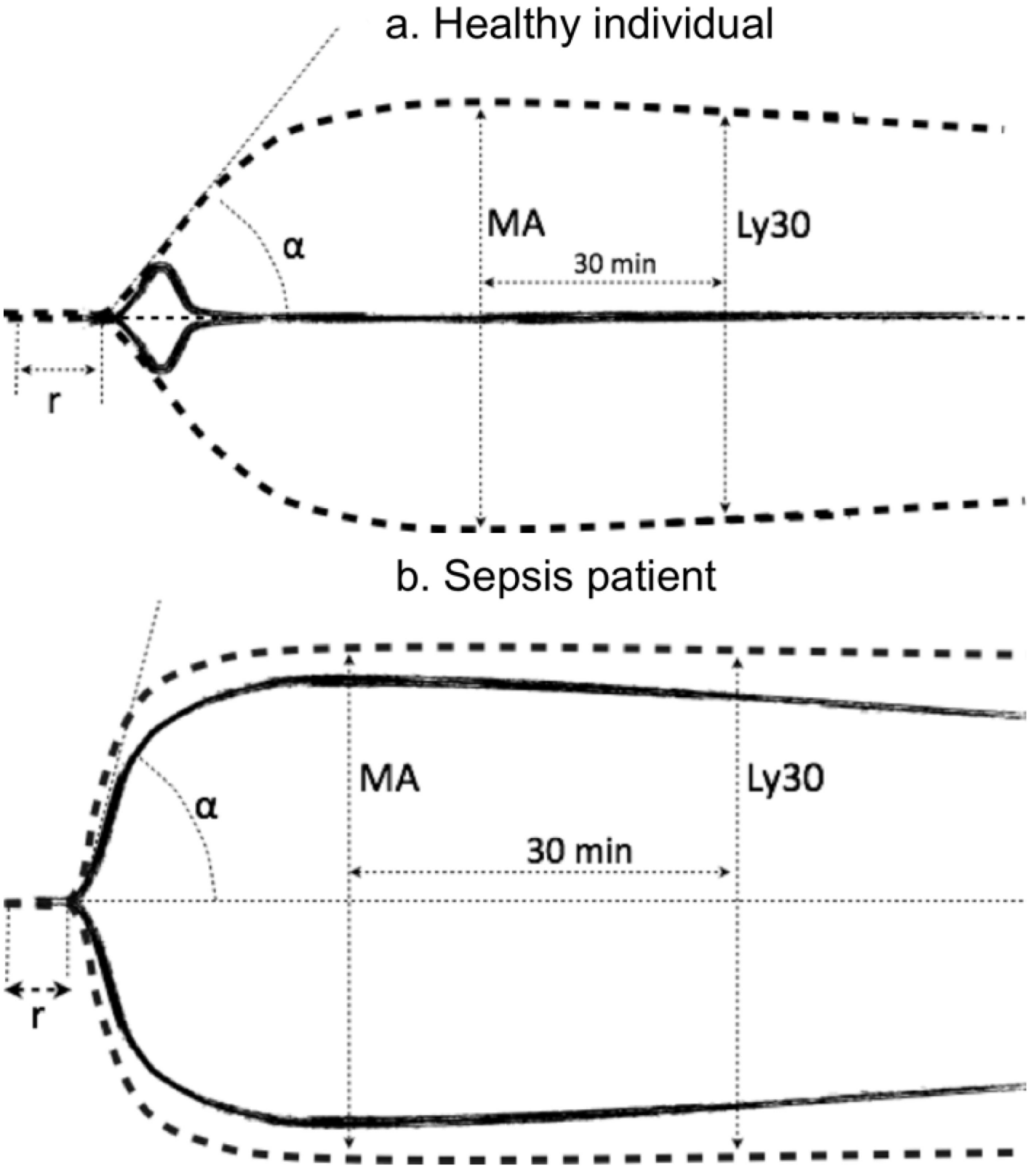
Graphical representation of two typical thromboelastographic tracings and parameters in a healthy individual (a) and a sepsis patient (b). TEG (dashed line) and UK-TEG (solid line) performed adding UK at a concentration of 80 IU/mL Abbreviations: r, reaction time; α, angle α; MA, maximal amplitude; Ly30, lysis at 30 min from MA.

UK concentration to perform UK-TEG procedure was assessed in a preliminary phase (S1 and S2 Figs). To ensure a fibrinolytic response, the UK concentration was set to 80 IU/ml. As shown in Fig 1, the typical TEG profile after the addition of urokinase (solid line) is characterized by: (i) an increase in Ly30 value, because of the increased conversion of plasminogen in plasmin and consequent increase rate of degradation of clot, (ii) decrease in MA, because the rate of clot breakdown becomes higher than the rate of clot formation.

Reproducibility of UK-TEG_Ly30 values at a UK concentration of 80 IU/ml was confirmed for different UK dilutions (S3 Fig) and for storage conditions of reagent (fresh, immediately reconstituted UK solution compared to -80°C frozen aliquots of UK solution rewarmed at room temperature before utilization, unpublished data).

Heparinase cups were used for TEG procedures. Although aware of the potential artifactual hyperfibrinolysis using heparinase cup [29] [30] we preferred to exclude potential effects of heparin on coagulation (S4 Fig). As a preliminary analysis of 203 K-TEG samples showed that UK-TEG_Ly60 could be predicted from UK-TEG_Ly30 (y = 1.3355+3.6525*1-exp(-0.0025*x), r^2^ = 0.99), we decided to used only UK-TEG_Ly30 values to assess response to UK in order to limit procedure execution times (S5 Fig).

Limits of normality (defined as the prediction interval between which 95% of values of healthy individuals group fall into, and calculated as: normal range = media ± 1.96*SD) were calculated on 40 healthy individuals and were 0–6.7% for TEG_Ly30 and 64.9–100% for UK-TEG_Ly30.

### PAI-1, TAFI, D-Dimer

Platelet poor plasma was obtained by centrifugation of citrated blood at 2500 g for 20 min at room temperature. Aliquots of citrated plasma, 1 ml each, were stored, shortly after collection, at -80°C until PAI-1 antigen, PAI-1 activity, TAFI assay were performed.

D-dimer concentrations were measured in citrated plasma by the d-dimer HS kit on ACL TOP analyzer (Instrumentation Laboratory, IL, USA). PAI-1 antigen was analyzed in duplicate by the ELISA kit ZYMUTEST PAI-1 Antigen (HYPHEN Biomed France). PAI-1 activity was measured in duplicate by the ELISA kit ZYMUTEST PAI-1 Activity (HYPHEN Biomed France). TAFI antigen was analyzed in duplicate by the IMUCLONE TAFI ELISA (Sekisui Diagnostics USA).

## Statistical Analysis

Continuous variables were described using median and 25^th^ and 75^th^ percentile. Absolute and relative frequencies were used for categorical variables. Differences between groups were tested using Mann–Whitney or Kruskal-Wallis rank test followed by post-hoc multiple comparison analyses (Dunn’s test with Bonferroni correction). Fisher’s exact test was used for categorical data. Correlations were expressed using the Spearman correlation coefficient. Cohen’s kappa coefficient was used to measure agreement between ISTH 2001 “overt” and JAAM 2006 DIC scores. We used univariate logistic regression to calculate odds ratios (ORs) and 95% confidence intervals (95% CIs) of death at ICU discharge in association with laboratory and TEG lysis parameters. Predicted risk of death (%) was then calculated using the formula: p = odds/(1 + odds)x100. Statistical analysis was performed using Stata 13; StataCorp, College Station, TX. We considered p < 0.05 to be statistically significant.

## Results

### Patients

Characteristics of patients and healthy individuals are reported in Table 1. Forty consecutive critically ill patients with severe sepsis or septic shock admitted to ICU were included in the study. Severe sepsis was diagnosed in 23 (58%) patients and septic shock in 17 (42%) patients. Twenty-one (52%) patients required vasopressor infusion at the time of blood sampling. Among the 40 sepsis patients, 5 (12%) had overt disseminated intravascular coagulation (DIC) defined according to the ISTH “overt” DIC score [26] and 21 (52.5%) according to the JAAM 2006 Score [27]. Thirty-two patients (80%) survived at ICU discharge and 8 (20%) died. Only one patient was receiving heparin prophylaxis. Healthy individuals were younger than patients (43 years (30–55) vs 61 years (49–76), p<0.001). The origin site of sepsis was identified as pulmonary in 23 (58%) of the patients, abdominal in 14 (35%), soft tissue in 2 (5%) and nervous system (meningitis) in 1 (2.5%). Microorganisms responsible of sepsis were identified in 23 patients. 14 patients were infected by gram-positive cocci, 4 patients by Enterobacteriae, 4 by other gram-negative bacteria and 1 by a co-infection of H1N1 influenza A virus and Pneumocystis Carinii. Prior surgical interventions were performed in 15 patients. Ten patients underwent abdominal surgery, three patients underwent thoracic surgery and two patients head and neck surgery.

**Table 1:** Characteristics of healthy individuals and sepsis patients.

	Healthy Individuals (N = 40)	Sepsis Patients (N = 40)	P value*
**Age (years)**	43 (30–55)	61 (49–76)	<0.001
**Males, N (%)**	18 (45)	24 (60)	0.18
**Cancer, N (%)**	-	1 (2.5)	-
**Smoking, N (%)**	12 (30)	8 (20)	0.44
**Severe sepsis, N (%)**	-	23 (58)	-
**Septic shock, N (%)**	-	17 (42)	-
**SOFA at admission**	-	7 (4–10)	-
**DIC (ISTH), N (%)**	-	5 (12.5)	-
**DIC (JAAM), N (%)**	-	21 (52.5)	-
**Mortality, N (%)**	-	8 (20)	-
**Timing between onset of sepsis and sampling (h)**	-	12 (6–24)	-
**Subjects on heparin, N (%)**	-	1 (2.5%)	-
**Site of infection**			
Pulmonary, N (%)	**-**	23 (57.5)	-
Abdominal, N (%)	-	14 (35)	-
Soft tissue, N (%)	-	2 (5)	-
Meningitis, N (%)	-	1 (2.5)	-
**Microorganism**			
Gram-positive cocci	-	14 (35)	-
Enterobacteriae	-	4 (10)	-
Other gram-negative bacilli	-	4 (10)	-
Virus/Pneumocystis carinii	-	1 (2.5)	-
Unknown	-	17 (42.5)	-

*From Mann-Whitney (continuous variables) or Fisher’s exact test (categorical variables).

Numbers in table refer to median (25^th^– 75^th^) or N(%). Abbreviations: SOFA, Sequential Organ Failure Assessment; DIC, disseminated intravascular coagulation; ISTH, International Society of Thrombosis and Haemostasis; JAAM, Japanese Association for Acute Medicine. Gram-positive microorganisms were: Methicillin Resistant Staphilococcus Aureus, Methicillin Subsceptible Staphilococcus Aureus, Enterococcus, Streptococcus Pyogenes, Streptococcus Pneumoniae. Enterobacteriae were: Escherichia Coli, Enterobacter, Serratia Marcescens. Other gram-negative bacilli were: Acynetobacter, Legionella Pneumophila. Virus was H1N1 influenza A virus.

Sepsis patients were characterized by a prolonged PT ratio (1.36 (1.20–1.54) vs 1.06 (1.01–1.10, p<0.001) and aPTT ratio (1.14 (1.03–1.29) vs 1.04 (0.98–1.07), p = 0.01, and by an higher fibrinogen (533 (380–704) vs 253 (244–283) mg/dl, p<0.001) and d-dimer levels (2090 (892–5602) vs 79 (60–127) ng/ml, p<0.001), compared to healthy individuals. Platelet count in sepsis patients was 138 (96–248) x10^9^/l. Sepsis patients had also an increase of PAI-1 antigen (31.7 (21.2–41.6) vs 3.3 (1.5–5.1) ng/ml, p<0.0001) and PAI-1 activity (4.0 (1.0–9.9) vs 0.2 (0.1–0.3) ng/ml, p<0.001) compared to healthy individuals. No significant differences were observed in TAFI values. Septic shock patients were characterized by higher fibrinogen (439 (370–479) vs 659 (487–749) mg/dl, p<0.05) and lower d-dimers levels (2760 (2018–6170) vs 1153 (746–5034) ng/ml, p<0.05) compared to severe sepsis patients. No statistically significant differences were observed among other coagulation parameters (S1 Table).

### TEG and UK-TEG

Fig 1 shows a graphical explanation of distinct thromboelastographic parameters as used in the current study and a typical example of the effect of UK on the TEG assay.

**Fig 2:**
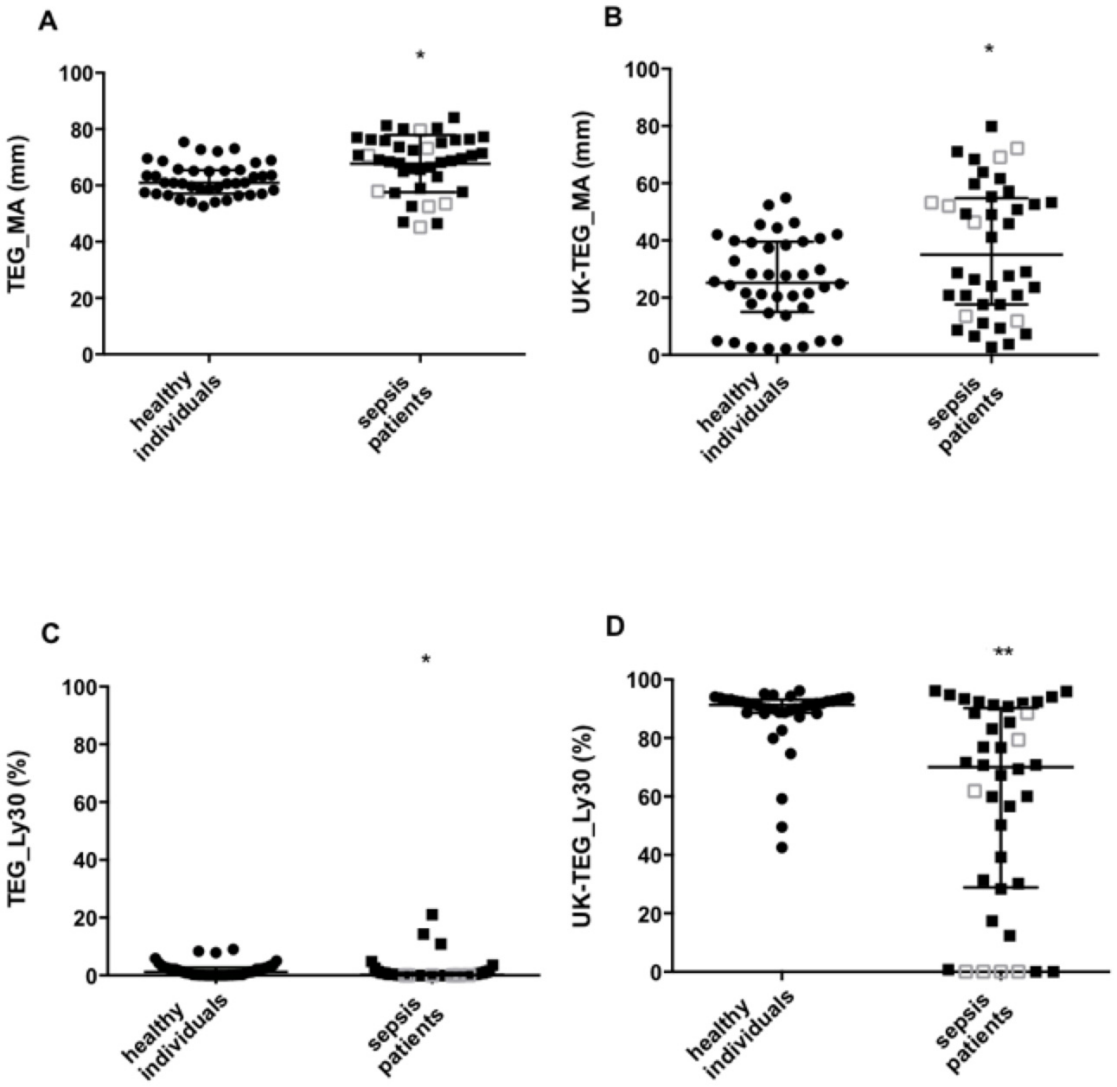
Distribution of MA and Ly30 measured by TEG and UK-TEG in healthy individuals and sepsis patients. Column scatter-plot for TEG_MA (Panel A), UK-TEG_MA (Panel B), TEG_Ly30 (Panel C) and UK-TEG_Ly30 (Panel D) in healthy individuals (circles) and sepsis patients (squares). Filled squares represent sepsis patients with SOFA score ≤ 10 (lower severity of disease), clear squares represent sepsis patients with SOFA score > 10 (higher severity of disease). Horizontal lines represent median (25^th^- 75^th^). * p<0.05, *** p<0.001, p value from Mann-Whitney test. TEG_MA, MA measured by TEG, UK-TEG_MA, MA measured by UK-TEG, TEG_Ly30, Ly30 measured by TEG, UK-TEG_Ly30, Ly30 measured by UK-TEG.

Both TEG and UK-TEG showed an ex-vivo impairment of fibrinolysis in sepsis patients compared to healthy individuals. TEG_MA was higher and TEG_Ly30 was lower in sepsis patients compared to healthy individuals (69 (60–76) vs 61 (57–65) mm, p = 0.001 and 0.1 (0–0.9) vs 1.2 (0.1–2.7) %, p = 0.007, respectively). Other TEG parameters were not different between the two groups. The addition of UK to TEG analysis resulted in a more pronounced difference between the two populations, UK-TEG_MA was higher (35 (18–55) vs 25 (15–39) mm, p = 0.026) and UK-TEG_Ly30 was lower (70 (29–90) vs 91 (89–93) %, p<0.0001) in sepsis patients compared to healthy individuals (Fig 2). The impairment of fibrinolysis was highly variable within the sepsis population, ranging from near normal to severely impaired fibrinolysis. Other UK-TEG parameters were not different between the two groups. No differences in TEG and UK-TEG parameters were observed between severe sepsis and septic shock groups.

### Correlations between Different Fibrinolysis Parameters

**Fig 3:**
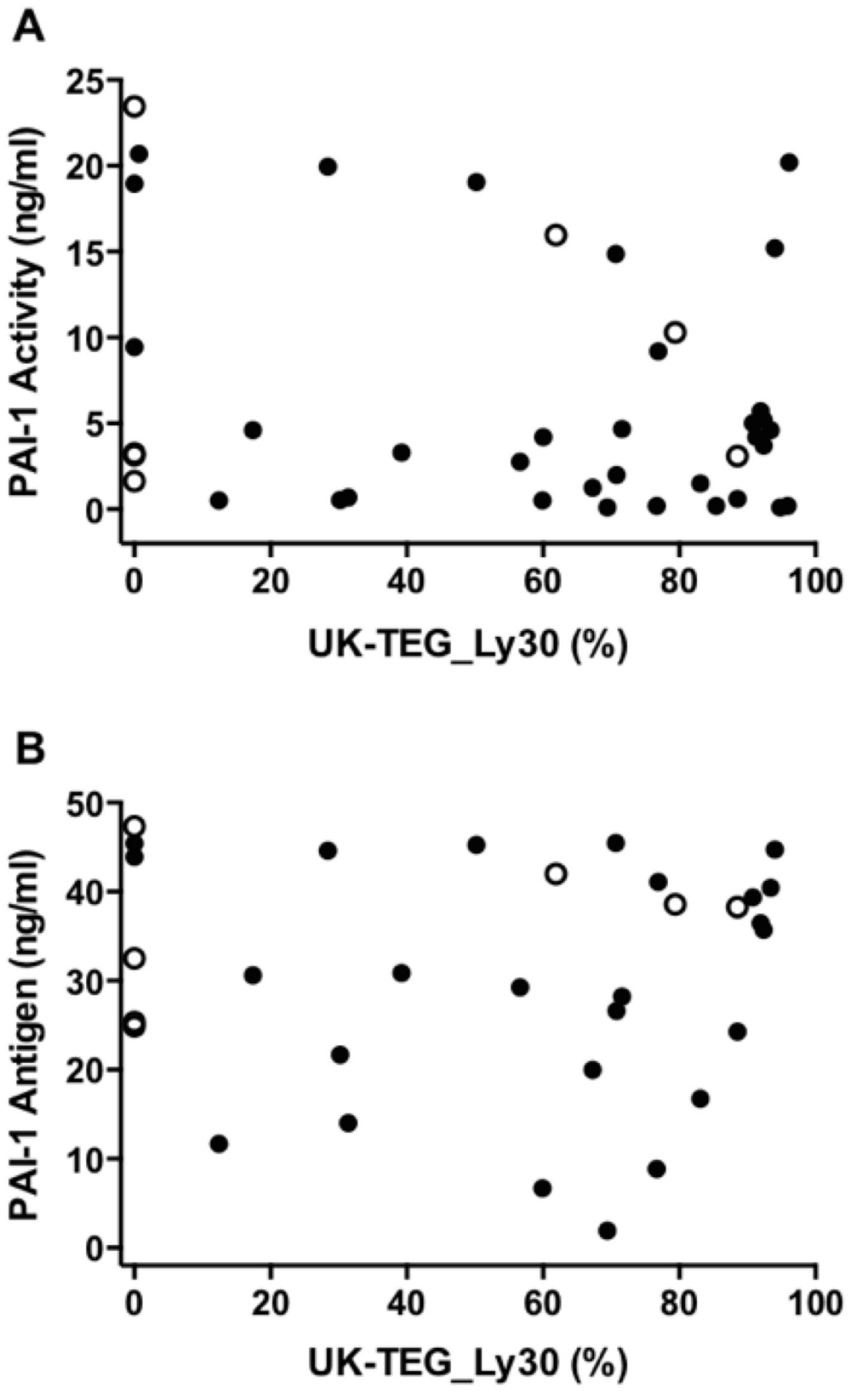
Correlation between different methods for assessing fibrinolysis in sepsis patients. Panel A: Correlation between PAI-1 Activity (ng/ml) and UK-TEG_Ly30 (%), Spearman's rho = -0.13, p = 0.44. Panel B: Correlation between PAI-1 Antigen (ng/ml) and UK-TEG_Ly30 (%), Spearman’s rho = -0.06, p = 0.72. Filled circles represent sepsis patients with SOFA score ≤ 10 (lower severity of disease), clear circles represent sepsis patients with SOFA score > 10 (higher severity of disease). PAI-1, Plasminogen Activator Inhibitor 1; UK-TEG_Ly30, Ly30 measured by UK-TEG.

Fig 3 shows correlation between PAI-1 and UK-TEG. UK-TEG_Ly30 was not correlated either with PAI-1 activity or with PAI-1 antigen (Spearman’s rho = -0.13, p = 0.44 and Spearman’s rho = -0.06, p = 0.72, respectively). UK-TEG_Ly30 was not correlated with d-dimer (Spearman’s rho = -0.07, p = 0.69). PAI-1 activity was significantly correlated with PAI-1 antigen (Spearman’s rho = 0.93, p<0.00001) as expected. There was no significant correlation among the remaining examined laboratory markers of fibrinolysis (d-dimer vs PAI-1 activity, Spearman’s rho = -0.16, p = 0.31; d-dimer vs PAI-1 antigen Spearman’s rho = -0.09, p = 0.59; d-dimer vs TAFI, Spearman’s rho = -0.02, p = 0.90; PAI-1 activity vs TAFI, Spearman’s rho = 0.05, p = 0.77; PAI-1 antigen vs TAFI, Spearman’s rho = 0.01, p = 0.97).

### Stratification of Patients According to the Degree of Hypofibrinolysis at UK-TEG

When patients were divided according to UK-TEG_Ly30 limits of normality, 22 (55%) were classified as “normal responders” (who exhibited a response to UK not different from healthy individuals) and 18 (45%) as “low responders” (who exhibited a response to UK lower than healthy individuals). As shown in Table 2, patients with low response to UK had more severe organ dysfunction (SOFA score 8 (6–13) vs 5 (4–7), p = 0.03), higher lactate levels (1.8 (1.2–2.7) vs 1.0 (0.8–1.9) mmol/L, p = 0.03), higher LDH (520 (388–1071) vs 374 (252–458) U/L, p = 0.01) and higher mortality (7(39%) vs 1(5%), p = 0.01) compared to those with a normal response, despite similar mean arterial pressure, arterial blood hemoglobin concentration and oxygen saturation. Prevalence of DIC was 5 (28%) in low responders compared to 0 in normal responders (p = 0.03) using the ISTH “overt” DIC score, and 10 (55.5%) in low responders vs 11 (50%) in normal responders (p = 0.5) when the JAAM 2006 DIC score was used. Time elapsed from diagnosis of sepsis and blood sampling was not different between low responders compared to normal responders (12 (6–24) vs 12 (3–12) hours, p = 0.61). Overall blood product administration and specifically fresh frozen plasma administration before blood sampling was not associated with response to UK at TEG analysis (p = 1.0 and p = 0.35, respectively). Incidence of ARDS or Acute Kidney Injury was not associated with response to UK at TEG analysis (p = 1.0 and p = 0.30, respectively) (S2 Table). Site of infection and microorganism genus were not associated with response to UK at TEG analysis (p = 1.00 and p = 0.96, respectively) (S3 Table).

**Table 2:** Clinical and laboratory parameters of sepsis patients in “normal response to UK” group and in “low response to UK” group.

	Normal response to UK (n = 22)	Low response to UK (n = 18)	P value*
SOFA	5 (4–7)	8 (6–13)	0.03
Lactate (mmol/L)	1.0 (0.8–1.9)	1.8 (1.2–2.7)	0.03
LDH (U/L)	374 (252–458)	520 (388–1071)	0.01
Creatinine (mg/dl)	0.85 (0.7–1.2)	1.50 (0.8–2)	0.09
Bilirubin	0.5 (0.4–0.9)	1.1 (0.7–1.5)	0.02
Platelet (10^3^/mmc)	169 (103–277)	131 (52–178)	0.14
DIC (ISTH), N (%)	0	5 (28)	0.01
DIC (JAAM), N (%)	11 (50)	10 (55)	0.76
MAP (mmHg)	81 (76–90)	78 (67–89)	0.35
Hb (g/dL)	9.9 (9.1–12.4)	11.1 (10.2–12.2)	0.20
SpO_2_ (%)	98 (95–100)	97 (95–99)	0.51
SvO2 (%)	72 (67–78)	71 (67–76)	0.68
Severe Sepsis/Septic Shock, N (%)	14 (64)/8 (36)	9 (50)/9 (50)	0.52
Hospital length of stay (days)	33 (15–44)	19.5(8–39)	0.20
Duration of mechanical ventilation (days)	5.5 (3–14)	4.5 (1–8)	0.35
ICU length of stay (days)	7 (4–20)	5 (3–9)	0.44
Mortality in ICU, N (%)	1 (5)	7 (39)	0.01

*P value from Mann-Whitney (continuous variables) or Fisher’s exact test (categorical variables). Numbers in table refer to median (25^th^– 75^th^) or N(%). Low response to UK group is defined as those patients with UK-TEG_Ly30 value less than 64.9% (limits of normality of healthy individuals UK-TEG_Ly30 = 64.9%-100%).

Abbreviations: SOFA, Sequential Organ Failure Assessment; LDH, lactate dehydrogenase; DIC, disseminated intravascular coagulation; MAP, mean arterial pressure; Hb, hemoglobin; ScvO_2_, central venous oxygen saturation; SpO2, pulse oximetry.

Blood product administration was not associated with response to UK at TEG analysis

As shown in Table 3, both patients with low response to UK and patients with normal response to UK had significantly increased levels of coagulation parameters, except the aPTT ratio and TAFI (%), compared to healthy individuals. Patients with low response to UK were characterized by a prolonged TEG_r (11.0 min (7.2–14.2) vs 7.5 min (6.1–10.5), p = 0.03) higher UK-TEG_MA (53.3 mm (49.0–68.4) vs 22.3 mm (9.4–29.0), p<0.0001) and lower UK-TEG_Ly30 (22.9% (0–50.2) vs 88.5% (76.7–92.4) compared to normal responders. Patients with normal response to UK differed significantly from healthy individuals only at TEG_MA (70.8 mm (66.5–77.0) vs 61.0 mm (57.3–65.4)).

**Table 3:** Coagulation and TEG parameters in healthy individuals, sepsis patients with “normal response to UK” and sepsis patients with “low response to UK”.

	Healthy Individuals	Sepsis Patients		P value*
		*Normal Response to UK*	*Low Response to UK*	
Coagulation Parameters	(N = 10)	(N = 22)	(N = 18)	
*PT ratio*	1.06 (1.01–1.10)	1.26 (1.12–1.42)^	1.46 (1.28–1.62)§§	<0.001
*aPTT ratio*	1.04 (0.98–1.07)	1.08 (1.01–1.26)	1.17 (1.07–1.31)	0.05
*Fibrinogen (mg/dl)*	252 (244–283)	565 (384–682)^^	529 (370–749)§§	<0.001
*D-dimer (ng/ml)*	78 (60–127)	1467 (810–6170)^^	2250 (1182–3472)§§	<0.001
*PAI-1 antigen (ng/ml)*	3.3 (1.5–5.1)	36.1 (20–39.6)^^	30.7 (24.3–43.9)§§	<0.001
*PAI-1 activity (ng/ml)*	0.2 (0.1–0.3)	4.0 (0.6–5.7)^	3.8 (1.6–19.0)§§	<0.001
*TAFI (%)*	110 (93–124)	104 (83–130)	111 (91–133)	0.94
TEG Parameters	(N = 40)	(N = 22)	(N = 18)	
*TEG_r (min)*	9.1 (7.6–10.1)	7.5 (6.1–10.5)	11.0 (7.2–14.2) °	0.03
*TEG_angle (deg)*	55.9 (47.6–62.6)	59.6 (48.8–68.6)	51.3 (39.6–59.9)	0.16
*TEG_MA (mm)*	61.0 (57.3–65.4)	70.8 (66.5–77.0) ^^	66.9 (52.6–73.2) °	<0.001
*TEG_Ly30 (%)*	1.2 (0.1–2.7)	0.3 (0–1.4)	0.1 (0–0.9) §	0.02
*UK-TEG*				
*UK-TEG_r (min)*	8 (7.0–8.7)	7.6 (5.8–10.0)	11.3 (6.6–12.6)	0.06
*UK-TEG_angle (deg)*	54.7 (37.5–65.0)	49.4 (34.3–67.0)	42.3 (32.8–57.3)	0.40
*UK-TEG_MA (mm)*	25.2 (15.6–39.8)	22.3 (9.4–29.0)	53.3 (49.0–68.4) §§ °°	<0.001
*UK-TEG_Ly30 (%)*	91.3 (88.5–93.1)	88.5 (76.7–92.4)	22.9 (0–50.2) §§ °°	<0.001

*P value from Kruskal-Wallis rank test. Post-hoc multiple comparison analyses from Dunn’s test with Bonferroni correction,

^°^ low responders vs normal responders p<0.05;

^°°^ low responders vs normal responders p<0.001;

^§^ low responders vs healthy individuals p<0.05;

^§§^ low responders vs healthy individuals p<0.001;

^^^ normal responders vs healthy individuals p<0.05;

^^^^ normal responders vs healthy individuals p<0.001.

Numbers in table refer to median (25^th^– 75^th^). Low response to UK group is defined as those patients with UK-TEG_Ly30 value less than 64.9% (limits of normality of healthy individuals UK-TEG_Ly30 = 64.9%-100%).

Abbreviations: PT ratio, prothrombin time ratio; aPTT ratio, activated partial thromboplastin time ratio; PAI-1, Plasminogen Activator Inhibitor 1; TAFI, Thrombin Activatable Fibrinolysis Inhibitor; TEG, Thromboelastography; TEG_r, reaction time measured by TEG; TEG_angle, angle α measured by TEG; TEG_MA, maximum amplitude measured by TEG; TEG_Ly30, lysis at 30 minutes after MA measured by TEG; UK-TEG, urokinase kaolin activated thromboelastography, UK-TEG_r, reaction time measured by UK-TEG; UK-TEG_angle, angle α measured by UK-TEG; UK-TEG_MA, maximum amplitude measured by UK-TEG; UK-TEG_Ly30, lysis at 30 minutes after MA measured by UK-TEG.

### Odds Ratio of TEG and Biochemical Markers of Fibrinolysis in Predicting Mortality at ICU Discharge and Morbidity

**Fig 4:**
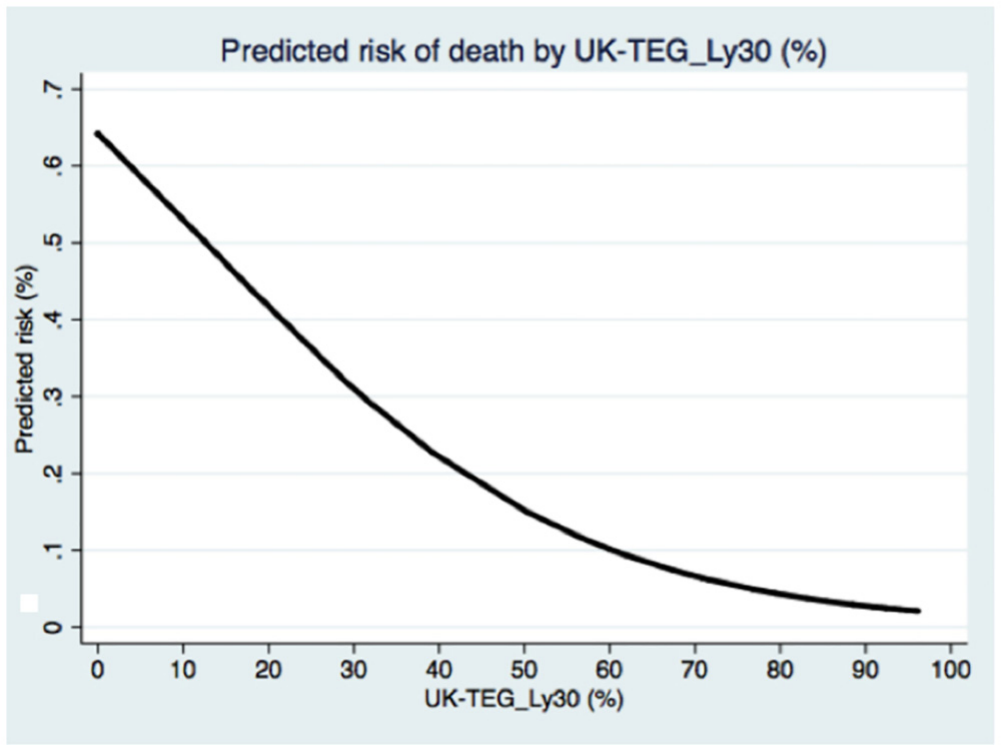
Predicted probability (risk) of death in relation with UK-TEG_Ly30 (%).

Table 4 summarizes odds ratio of TEG, UK-TEG, d-dimer, PAI-1 activity, PAI-1 antigen and TAFI for mortality at ICU discharge. Only UK-TEG_Ly30 could predict significantly mortality (OR 0.95, 95% CI 0.93–0.98, p = 0.003) at ICU discharge. Fig 4 shows predicted mortality risk according to UK-TEG_Ly30 values, the lower UK-TEG_Ly30 values, the higher the predicted risk of death in ICU.

**Table 4:** Odds ratios (OR) and 95% confidence interval (95% CI) of death for different lysis parameters using logistic regression analysis.

Lysis parameter	Odds ratio	95% CI	p value
D-dimer (10^**3**^ng/ml)	1.06	0.98–1.15	0.15
PAI activity (ng/ml)	1.08	0.98–1.20	0.13
PAI antigen (ng/ml)	1.07	0.99–1.16	0.11
TAFI (%)	1.00	0.98–1.02	0.89
TEG_Ly30 (%)	0.001	0.00–18.8	0.17
UK-TEG_Ly30 (%)	0.95	0.93–0.98	0.003

Table 5 summarizes odds ratio of TEG, UK-TEG, d-dimer, PAI-1 activity, PAI-1 antigen and TAFI for predicting higher severity (defined as SOFA>10). Only UK-TEG_Ly30 could predict significantly SOFA > 10 (OR 0.98, 95% CI 0.95–0.99, p = 0.049).

**Table 5:** Odds ratios (OR) and 95% confidence interval (95% CI) of having a SOFA score >10 (higher severity of disease) for different lysis parameters using logistic regression analysis.

Lysis parameter	Odds ratio	95% IC	p value
D-dimer (10^**3**^ng/ml)	1.00	0.99–1.00	0.09
PAI activity (ng/ml)	1.05	0.94–1.17	0.40
PAI antigen (ng/ml)	1.05	0.97–1.13	0.25
TAFI (%)	1.00	0.97–1.02	0.79
TEG_Ly30 (%)	0.001	0.00–491.03	0.23
UK-TEG_Ly30 (%)	0.98	0.95–0.997	0.049

UK-TEG_Ly30 was negatively correlated (p = 0.01, Spearman’s rho = -0.4) with SOFA score indicating less potential for fibrinolysis when increasing severity of the illness.

## Discussion

In this study we found: I. that the fibrinolysis assessment at the point of care is feasible; II. that only less than half of the patients presented ex-vivo impairment of fibrinolysis III. that this subgroup of patients was characterized by higher markers of cellular damage, higher severity score and worse outcome.

TEG is an established point of care technique used to guide transfusion management in trauma patients [31], cardiac surgery [32] or orthotopic liver transplantation [33] [34] and had been shown to be a prognostic factor in critically ill patients [35]. Unfortunately, as it is currently conceived, TEG cannot assess the degree of hypofibrinolysis. Numerous methods [36] [17] [37] [1] have been reported to investigate the ex-vivo fibrinolytic potential of the whole blood with viscoelastic hemostatic assays, although to our knowledge, none of them has been used to detect impaired fibrinolysis in sepsis patients. The modification that we made to the TEG technique (UK-TEG) was aimed at amplifying ex-vivo the fibrinolytic response. We observed that UK-TEG lysis index was lower in sepsis patients compared to controls, indicating a lower degree of fibrinolysis. As expected PAI-1 levels were higher in sepsis patients compared to controls, however they were unrelated to UK-TEG lysis index. This lack of correlation may be explained by the fact that PAI-1 is only one of the factors involved in the fibrinolytic process, the role of which may become relevant assuming that all the other factors are normal or at least homogenous. In our study, patients had very different levels of fibrinogen and possibly also of plasminogen, which unfortunately was not measured but is known to be reduced in severe sepsis/septic shock [38]. We also measured TAFI levels in plasma. TAFI modulates fibrinolysis removing the lysine residues from the fibrin clot and preventing its degradation by plasmin. Unexpectedly TAFI values were not different between sepsis patients and controls. It must be said, however, that we did not measure the upregulation of the TAFI pathway [39] and the lack of this information does not allow us to exclude an enhancement of the TAFI antifibrinolytic pathway.

Our goal was to use a global approach of hemostasis assessment via a point of care modified technique to quantify global fibrinolysis. We acknowledge however that fibrinolysis in the sepsis patient may be affected by several factors: I. a defect in the release of plasminogen activators by endothelial cells (tPA) or by leukocytes (UK), II. an increased concentration of inhibitors in the presence of normal release of plasminogen activators, III. a combination of these abnormalities (low plasminogen activators and increased inhibitors), IV. a very low plasminogen concentration. Due to lack of information about all these variables we are unable to speculate on the underlying mechanism.

More than half of the sepsis patients met the DIC criteria according to the JAAM 2006 score. Kappa coefficient between JAAM 2006 and ISTH 2001 “overt” score was 0.23 (fair) and only 5 patients with DIC were diagnosed by both scoring systems. All the patients who developed the ISTH DIC (overt DIC) could be identified based on the JAAM DIC criteria confirming previous findings [27, 40, 41]. In patients with defective fibrinolysis (according to response to UK), DIC was diagnosed more frequently only with the ISTH 2001 “overt” score. We may hypothesize that although the JAAM scoring system (differing from the ISTH by the inclusion of the systemic inflammatory response syndrome, higher cut-off in the platelet count, and exclusion of fibrinogen) has higher sensitivity for the diagnosis of early DIC [40] it could have lower specificity than the ISTH scoring system for detecting DIC with impairment of the fibrinolytic system.

According to UK-TEG, impairment of fibrinolysis was present only in 45% of the patients (and 35% according to PAI-1), confirming our previous findings [4]. Abnormal levels of fibrinolysis inhibitors such as PAI-1 and TAFI were observed in sepsis patients [42] [43] [10] and may value as a prognostic marker in septic shock [44] [11] [12] [45]. Most clinical trials investigating anticoagulant pharmacological strategies in severe sepsis and sepsis shock have so far failed to demonstrate a survival advantage [18], [19] in particular the promising results of the activated protein C (drotrecogin alfa-activated-, Xigris, Eli Lilly Inc) [46] had not been confirmed and its use was associated to an increased rate of hemorrhagic complications [47] [20] [21]. The use of recombinant human soluble thrombomodulin was observed to improve organ dysfunction in patients with sepsis-induced DIC [48] [49] but despite some evidence suggestive of efficacy, it still lacks of a mortality benefit [50]. With respect to the field of fibrinolysis, some promising results were reported when treatment with recombinant tissue-plasminogen activator was used to restore fibrinolysis in a pediatric population with meningococcemia [51], however the high risk of hemorrhagic adverse effects precluded the possibility of carrying out further studies. It is tempting to speculate that specific therapies guided by the early identification of the hypofibrinolytic patients could lead to a benefit.

The subgroup of patients with low response to the fibrinolytic stimulus (UK-TEG_Ly30 <65%) was characterized by worse coagulopathy, higher degree of cellular injury, higher SOFA score and mortality, compared to the subgroup of patients with normal response (Ly30 >65%) despite no evidence of inadequate systemic oxygen delivery (normal mean arterial pressure, normal arterial oxygen content, normal central venous oxygen saturation). Such a difference was not clearly evidenced at basal TEG. Moreover, UK-TEG lysis index was the only fibrinolytic marker able to predict mortality at ICU discharge and morbidity (rated with the SOFA score). The association between defective fibrinolysis and poor outcome suggests that, at least in this subgroup of patients, impairment of fibrinolysis may play an active pathogenic role altering microcirculation and contributing to multi-organ failure, further corroborating the hypothesis that in this framework a targeted pharmacologic intervention to “rescue” microcirculation, appears rationale.

We are aware that our study has some limitations. Only one time point was assessed. Timing of measurements may be relevant when assessing the coagulation status of a patient in ICU because coagulopathy in sepsis is a dynamic process. Performing sequential measurements would probably provide better insights. Also, our study, conceived as a pilot, was performed in a single center with a limited sample size, and requires confirmation in a larger population.

## Conclusion

Our results confirm that: I. UK-TEG is a feasible bedside tool to detect in real time sepsis-induced defective fibrinolysis, II. less than half of the patients showed ex-vivo impairment of fibrinolysis; III. this subgroup of patients was characterized by higher markers of cellular damage, higher severity score and worse outcome. Although UK-TEG lysis parameter was not correlated with standard laboratory markers of enhanced fibrinolysis, it was able to predict morbidity and mortality. These findings should be considered hypothesis generating for a targeted pharmacological approach to sepsis patients with low response to a fibrinolytic stimulus.

## Key Messages

- UK-TEG (Urokinase Kaolin activated Thromboelastography) is a feasible modified point-of-care global assay of hemostasis to assess fibrinolysis in sepsis patients
- Patients with defective fibrinolysis evidenced at UK-TEG were characterized by higher markers of cellular damage, higher morbidity and worse outcome.
- Early point of care identification of sepsis patients with defective fibrinolysis may be of future interest

## Supporting Information

S1 Fig Preliminary assessment of UK-TEG procedure. UK-TEG: titration curve for urokinase concentrations in whole blood.
(DOCX)

S2 Fig Preliminary assessment of UK-TEG procedure. Incremental concentrations of UK to assess the degree of response to the fibrinolytic stimulus in sepsis patients.
(DOCX)

S3 Fig Preliminary assessment of UK-TEG procedure. Reproducibility of two urokinase dilutions to obtain UK-TEG_Ly30 value at Urokinase concentration of 80 IU/ml.
(DOCX)

S4 Fig Preliminary assessment of UK-TEG procedure. Comparison between TEG_Ly30 using clear and heparinase cups.
(DOCX)

S5 Fig Preliminary assessment of UK-TEG procedure. Correlation between UK-TEG_Ly30 and UK-TEG_Ly60.
(DOCX)

S1 Table Coagulation and clinical parameters in severe sepsis/septic shock patients. (DOCX)

S2 Table Incidence of ARDS, Acute Kidney Injury and blood product administration in patients with normal and low response to UK. (DOCX)

S3 Table Site of infection and microorganisms responsible of sepsis in patients with normal and low response to UK. (DOCX)
