# Supplementary material for: Assessment of Fibrinolysis in Sepsis Patients with Urokinase Modified Thromboelastography
Source: PLoS One. 2015 Aug 26;10(8):e0136463. doi: 10.1371/journal.pone.0136463 (PMC4550424; doi:10.1371/journal.pone.0136463)
Supplement: S1 Fig — UK-TEG: titration curve for urokinase concentrations in whole blood. (DOCX) [file pone.0136463.s001.docx]

**S1 Figure**

**Preliminary assessment of UK-TEG procedure. UK-TEG: titration curve for urokinase concentrations in whole blood**

UK-TEG was preliminary assessed with three different concentrations (80 IU/ml, 120 IU/ml and 160 IU/ml) of urokinase in 17 healthy individuals and 17 randomly chosen critically ill patients (5 (28%) sepsis, 2 (11%) hemorrhagic shock, 2 (11%) lung transplantation, 2 (11%) COPD, 3 (16%) ARDS, 1 (6%) ischemic stroke, 2 (11%) pulmonary lobectomy and 1 (6%) cardiac arrest)). Nobody had pre-existing coagulation disorders. Data from these patients are not included in the present study.

A statistically significant difference in UK-TEG_Ly30 value between healthy individuals and critically ill patients was observed with at UK 160 IU/ml (89.46 ± 2.54 and 66.25 ± 9.16, respectively; p< 0.02).
