# Supplementary material for: Assessment of Fibrinolysis in Sepsis Patients with Urokinase Modified Thromboelastography
Source: PLoS One. 2015 Aug 26;10(8):e0136463. doi: 10.1371/journal.pone.0136463 (PMC4550424; doi:10.1371/journal.pone.0136463)
Supplement: S2 Fig — Incremental concentrations of UK to assess the degree of response to the fibrinolytic stimulus in sepsis patients. (DOCX) [file pone.0136463.s002.docx]

**S2 Figure**

**Preliminary assessment of UK-TEG procedure. Incremental concentrations of UK to assess the degree of response to the fibrinolytic stimulus in sepsis patients**

After a preliminary phase (Appendix, figure 1) the study was conducted only on sepsis patients and UK concentration initially set at 80 IU/ml and then incrementally adjusted (up to 160 and 240 IU/ml) if UK-TEG_Ly30 value at 80 IUI/ml was below 40% to reveal the cut-off to induce fibrinolysis.

A preliminary analysis of the first 12 sepsis patients revealed the same trend in all patients with a progressive increase of UK-TEG_Ly30 value i.e. UK-TEG_Ly30 at 80 IU/ml median 6 (0-30) %, UK-TEG_Ly30 at 160 IU/ml 54 (2-78) %, UK-TEG_Ly30 at 240 IU/ml 84 (74-92) %, ANOVA p= 0.0034.

On the contrary, all healthy individuals showed a UK-TEG_Ly30 value at 80 IU/ml above 40%, thus the study was conducted using a UK concentration of 80 IU/ml.
