# Supplementary material for: Assessment of Fibrinolysis in Sepsis Patients with Urokinase Modified Thromboelastography
Source: PLoS One. 2015 Aug 26;10(8):e0136463. doi: 10.1371/journal.pone.0136463 (PMC4550424; doi:10.1371/journal.pone.0136463)
Supplement: S3 Fig — Reproducibility of two urokinase dilutions to obtain UK-TEG_Ly30 value at Urokinase concentration of 80 IU/ml. (DOCX) [file pone.0136463.s003.docx]

**S3 Figure**

**Preliminary assessment of UK-TEG procedure. Reproducibility of two urokinase dilutions to obtain UK-TEG_Ly30 value at Urokinase concentration of 80 IU/ml**

Ten patients were tested to assess the reproducibility of the results of UK-TEG Ly30 at 80 IU/ml obtained with two different urokinase dilutions i.e. 20 IU/μl (total 4 μl added to 996 μl of citrated WB) and 4 IU/μl (total 20 μl added to 980 μl of citrated WB)

All of our data are in the range between -7.6 and +9.2% of the average of the Ly30 tested with two different urokinase dilutions, this finding suggests good agreement between UK-TEG_Ly30 using 4 IU/μl and 20 IU/μl of urokinase. Using urokinase 4 IU/μl we overestimated TEG_Ly30% of 0.8%.
