# Supplementary material for: Assessment of Fibrinolysis in Sepsis Patients with Urokinase Modified Thromboelastography
Source: PLoS One. 2015 Aug 26;10(8):e0136463. doi: 10.1371/journal.pone.0136463 (PMC4550424; doi:10.1371/journal.pone.0136463)
Supplement: S4 Fig — Comparison between TEG_Ly30 using clear and heparinase cups. (DOCX) [file pone.0136463.s004.docx]

**S4 Figure**

**Preliminary assessment of UK-TEG procedure. Comparison between TEG_Ly30 using clear and heparinase cups**

When testing clear (citrated kaolin, CK) and heparinase (citrated kaolin heparinase, CKH) cups to verify potential intraprocedural artifacts, our findings confirmed previous reports with a systematic TEG_Ly30 lower value in samples analyzed with clear cups compared to heparinase cups.

Most of our data are in the range between -37 and +26% of the average of the Ly30 tested with two different cups, this finding suggests good agreement between UK-TEG_Ly30 using clear and heparinase cups. Using clear cups we underestimated TEG_Ly30% of -5%.
