# Supplementary material for: Assessment of Fibrinolysis in Sepsis Patients with Urokinase Modified Thromboelastography
Source: PLoS One. 2015 Aug 26;10(8):e0136463. doi: 10.1371/journal.pone.0136463 (PMC4550424; doi:10.1371/journal.pone.0136463)
Supplement: S5 Fig — Correlation between UK-TEG_Ly30 and UK-TEG_Ly60. (DOCX) [file pone.0136463.s005.docx]

**S5 Figure**

**Preliminary assessment of UK-TEG procedure. Correlation between UK-TEG_Ly30 and UK-TEG_Ly60**

Ly60% is predicted by Ly30% according to an exponential function, f= 1.3355+3.6525*1-exp(-0.0025*x), r^2^ = 0.99.
