# Supplementary material for: Assessment of Fibrinolysis in Sepsis Patients with Urokinase Modified Thromboelastography
Source: PLoS One. 2015 Aug 26;10(8):e0136463. doi: 10.1371/journal.pone.0136463 (PMC4550424; doi:10.1371/journal.pone.0136463)
Supplement: S1 Table — (DOCX) [file pone.0136463.s006.docx]

**S6 Table**

**Coagulation and clinical parameters in severe sepsis/septic shock patients**

|  | **SEVERE SEPSIS**  **(N= 23)** | **SEPTIC SHOCK (N=17)** | **p value** |
| --- | --- | --- | --- |
| **Coagulation Parameters** | | | |
| *Platelet (10^3^/mmc)* | 173 (103-277) | 132 (78-178) | 0.20 |
| *PT ratio* | 1.28 (1.11-1.51) | 1.42 (1.25-1.62) | 0.07 |
| *aPTT ratio* | 1.10 (1.02-1.25) | 1.18 (1.07-1.30) | 0.22 |
| *Fibrinogen (mg/dl)* | 659 (487-749) | 439 (370-479) | 0.03 |
| *D-dimer (ng/ml)* | 1153 (746-5034) | 2760 (2018-6170) | 0.04 |
| *PAI-1 antigen (ng/ml)* | 30.6 (11.7-41.1) | 36.5 (24.3-43.4) | 0.40 |
| *PAI-1 activity (ng/ml)* | 4.2 (0.5-9.2) | 3.7 (1.6-14.9) | 0.45 |
| *TAFI (%)* | 115 (96-133) | 98 (85-124) | 0.29 |
| **TEG** | | | |
| *TEG_r (min)* | 7.8 (6.3-11.7) | 10.3 (7.1-12.8) | 0.16 |
| *TEG_angle (deg)* | 59.9 (42.4-67.2) | 49.5 (39.8-58.7) | 0.18 |
| *TEG_MA (mm)* | 71.4 (65.6-77.0) | 68.2 (57.9-70.7) | 0.09 |
| *TEG_Ly30 (%)* | 0.3 (0-0.9) | 0 (0-0.8) | 0.46 |
| **UKIF-TEG** | | | |
| *UKIF-TEG_r (min)* | 8.2 (5.8-10.8) | 9.2 (6.5-12.0) | 0.49 |
| *UKIF-TEG_angle (deg)* | 46.8 (32.8-61.1) | 42.6 (35.2-64.1) | 0.99 |
| *UKIF-TEG_MA (mm)* | 29.0 (11.1-57.2) | 45.9 (17.6-52.6) | 0.97 |
| *UKIF-TEG_Ly30 (%)* | 71.6 (39.2-91.3) | 60 (0.7-83.1) | 0.19 |
| **Clinical parameters** | | | |
| *ScvO2 (%)* | 74 (68-80) | 68 (63-75) | 0.07 |
| *Lactate (mmol/L)* | 1.2 (0.9-1.9) | 1.7 (0.9-3.3) | 0.18 |
| *LDH (IU/L)* | 424 (262-520) | 489 (331-889) | 0.36 |
| *Creatinine (mg/dL)* | 0.9 (0.7-1.5) | 1.3 (0.8-2.2) | 0.12 |
| *Bilirubin (mg/dL)* | 0.6 (0.4-1.0) | 1.1 (0.7-1.9) | 0.016 |
| *SOFA score* | 5 (4-7) | 9 (7-12) | <0.001 |
| *DIC score (ISTH)* | 3 (2-3) | 3 (3-4) | 0.09 |
| *DIC score (JAAM)* | 3 (2-5) | 5 (3-6) | 0.02 |
| *Mortality in ICU, N (%)* | 3 (13) | 5 (29) | 0.25 |
