# Supplementary material for: Assessment of Fibrinolysis in Sepsis Patients with Urokinase Modified Thromboelastography
Source: PLoS One. 2015 Aug 26;10(8):e0136463. doi: 10.1371/journal.pone.0136463 (PMC4550424; doi:10.1371/journal.pone.0136463)
Supplement: S2 Table — (DOCX) [file pone.0136463.s007.docx]

**S8 Table**

**Incidence of ARDS, Acute Kidney Injury and blood product administration in patients with normal and low response to UK**

|  | NORMAL RESPONSE to UK (n=22) | LOW RESPONSE  to UK (n=18) | P value |
| --- | --- | --- | --- |
| ARDS, N (%) | 2 (9) | 2 (11) | 1.0 |
| Acute Kidney Injury*, N (%) |  |  | 0.30 |
| *Stage 1* | 5 (23) | 3 (18) |  |
| *Stage 2* | 2 (9) | 4 (23) |  |
| *Stage 3* | 2 (9) | 4 (23) |  |
| Subjects transfused before the study, N (%)^ | 6 (26) | 4 (22) | 1.0 |

*Defined according to Acute Kideny Injury Network (AKIN) citeria [Mehta RL et al. Crit Care 2007;11(2):R31.]; ^ p=0.35 for fresh frozen plasma transfusions
