# Supplementary material for: Assessment of Fibrinolysis in Sepsis Patients with Urokinase Modified Thromboelastography
Source: PLoS One. 2015 Aug 26;10(8):e0136463. doi: 10.1371/journal.pone.0136463 (PMC4550424; doi:10.1371/journal.pone.0136463)
Supplement: S3 Table — (DOCX) [file pone.0136463.s008.docx]

**S7 Table**

**Site of infection and microorganisms responsible of sepsis in patients with normal and low response to UK**

|  | NORMAL RESPONSE to UK (n=22) | LOW RESPONSE  to UK (n=18) | P value |
| --- | --- | --- | --- |
| Site of Infection, N (%) |  |  | 1.00 |
| *Abdominal* | 8 (36) | 6 (33) |  |
| *Pneumonia* | 12 (54) | 11 (61) |  |
| *Meningitis* | 1 (5) | 0 |  |
| *Soft tissue* | 1 (5) | 1 (6) |  |
| Microorganism, N (%) |  |  | 0.96 |
| *Gram-positive cocci* | 8 (20) | 6 (15) |  |
| *Enterobacteriae* | 2 (5) | 2 (5) |  |
| *Other gram-negative*  *bacilli* | 2 (5) | 2 (5) |  |
| *Virus* | 0 | 1 (2.5) |  |
| *Unknown* | 10 (25) | 7 (17.5) |  |
